# Cytotoxin antibody-based colourimetric sensor for field-level differential detection of elapid among big four snake venom

**DOI:** 10.1371/journal.pntd.0009841

**Published:** 2021-10-11

**Authors:** Sherin Kaul, L. Sai Keerthana, Pankaj Kumar, Komal Birader, Yathirajarao Tammineni, Deepali Rawat, Pankaj Suman

**Affiliations:** Animal Biotechnology Laboratory, National Institute of Animal Biotechnology, Hyderabad, India; Muséum National d’Histoire Naturelle, FRANCE

## Abstract

Development of a rapid, on-site detection tool for snakebite is highly sought after, owing to its clinically and forensically relevant medicolegal significance. Polyvalent antivenom therapy in the management of such envenomation cases is finite due to its poor venom neutralization capabilities as well as diagnostic ramifications manifested as untoward immunological reactions. For precise molecular diagnosis of elapid venoms of the big four snakes, we have developed a lateral flow kit using a monoclonal antibody (AB1; IgG_1_ – κ chain; Kd: 31 nM) generated against recombinant cytotoxin-7 (rCTX-7; 7.7 kDa) protein of the elapid venom. The monoclonal antibody specifically detected the venoms of *Naja naja* (p < 0.0001) and *Bungarus caeruleus* (p<0.0001), without showing any immunoreactivity against the viperidae snakes in big four venomous snakes. The kit developed attained the limit of quantitation of 170 pg/μL and 2.1 ng/μL in spiked buffer samples and 28.7 ng/μL and 110 ng/μL in spiked serum samples for detection of *N*. *naja* and *B*. *caeruleus* venoms, respectively. This kit holds enormous potential in identification of elapid venom of the big four snakes for effective prognosis of an envenomation; as per the existing medical guidelines.

## Author summary

Detection of the snake species responsible for the envenomation in a victim is crucial for clinical and forensic management of poisoning cases. Polyvalent antivenom therapy can seldom lead to immunological complications manifested in the form of symptoms ranging from serum sickness to myalgia. Use of monovalent antivenom therapy is being recommended for targeted venom neutralization in envenomed individuals with minimum side effects. Moreover, the development of field applicable venom detection devices is the need of the hour for enabling orderly detection of poisoning cases at the crime scene, besides testing for illegal trade of snake parts, including venom, protected under the Wildlife Act. The monovalent antivenom therapy and the field level detection of venom conducive to adequate crime scene management is limited by the tools available for species or family-specific identification of the venom under question. For differential detection of the Elapids of the big four snakes from the Viperidae, we have developed a monoclonal antibody-based lateral flow assay kit using recombinant Cytotoxin– 7 protein. The limit of quantitation for the detection of venoms of *N*. *naja* and *B*. *caeruleus* was ascertained to be 170 pg/μL and 2.1 ng/ μL in spiked buffer samples and 28.7 ng/ μL and 110 ng/ μL in spiked serum samples, respectively. Thus, the kit can effectively detect the venoms of elapids of the big four snakes in both simple and complex matrices of the samples and can be adapted for its use in differential diagnosis.

## Introduction

Envenomation due to snakebite has long been declared as a Category “A” Neglected Tropical Disease [1] affecting >5 million people, with the death of ~100,000 people, and several others surviving with a consequential permanent disability, across the globe [2]. The prevalence of such incidences is profound across all continents especially in the tropical countries (Asia, Africa, Latin America, and Oceania) of the developing world, owing to their lifestyle. Among these countries, India acts as a hotspot for snake envenomation with almost 1.2 million deaths reported in the past two decades, majorly in the rural parts of the country [3]. Despite the reported ~3000 species of snakes, several are poisonous whereas many others are medically relevant due to their clinical implications. Among all, the “big four” snakes including *Naja naja*, *Bungarus caeruleus*, *Echis carinatus*, and *Daboia russelii* are primarily accountable for half of the deaths reported due to snakebite in India [4].

Management of snake bite cases mainly relies on timely administration of polyvalent antivenom raised in horses. Studies have shown that wherever available, monovalent antivenoms have a better target specificity and venom clearance ability making them the preferred choice in potentially fatal cases over the prevalent polyvalent antivenoms [5]. Despite the available findings, the monovalent antivenom therapy is not in practice due to the failure in the detection of specific causative species of snakes in cases of envenomation [6–8]. Due to polyvalent nature of existing antivenom used in therapy; large volume of antisera is administered to neutralize the venom in the body fluid leading to sensitization of the immune system to trigger extravagant immune reactions leading to different forms of permanent deformities like, disability of the affected organ, localized or systemic immunological complications and multiple organ failure [9]. It has seldom been reported that the identification of snake species based solely on the victim’s account, captured snake, or the visible clinical symptoms remain a challenge and can often be misinterpreted, resulting in detrimental consequences over inefficient victim management [10]. High precision in the diagnosis of the specific venom is mandatory to recommend monovalent anti-snake venom therapy to the victim(s). A wrong prescription due to improper diagnosis may lead to fatal consequences.

Identification of venom is also an issue for forensic science experts dealing with medicolegal cases suspected of poisoning and illegal trading of venom. Discerning the snake species from a variety of dried samples procured as evidence from any crime scene provides crucial links which substantiate the cause of death of the victim and even can help prevent insurance frauds. Hitherto, the analysis of this evidence relies on traditional techniques such as double diffusion assay, ELISA [11], or mitochondrial DNA analysis of the venom sample [12]. These traditional laboratory-based assays can seldom surpass the critical window of examination of the sample before it enters the victim circulation. The sensitive mt-DNA based detection assays are useful only in cases where the snake/venom (in pure form) is available for testing. Thus, to facilitate forensic examination, the need to develop a point-of-care venom detection kit has long been emphasized.

The past decade has seen an uprising in the genomic, proteomic, and mass spectrometric studies of the crude venom complex [13–16]. These studies have led to a deeper insight into the venomics of these samples for subsequent identification of the abundantly available proteins specific to the respective genus or species’ venoms responsible for inducing the clinical symptoms of lethality as reported [17–20]. The identification of such protein complexes or their families is beneficial for its utilization in the continual development of strategies for detection of the snake species from the venom samples. Traditionally, diagnosis for snake envenomation relied primarily on visual examination followed by polyclonal sera–based detection strategies such as, immunodiffusion, immunofluorescence, hemagglutination, immunoelectrophoresis, radioimmunoassay; which in turn were limited by their tendency to cross-react with the closely related venoms, the reduced assay sensitivity, poor scalability for industrial production, and a prolonged-time of detection [21–23]. With advancing technologies, portable ELISA based kits, optical immunoassay, dot-blot assay, and family-specific protein identification tests were developed for detection of the specific venom using a three-step affinity-purified polyclonal antibody [24–28]. Affinity-purified polyclonal antibody raised against a cocktail of selective venoms is limited by its scalability for industrial production. Large–scale production of polyclonal antibodies is expensive as well as liable for batch–to–batch variation in recognition of epitopes. Despite the attempts made for miniaturization of conventional assays with enhanced sensitivity, its field applicability remains questionable due to the requirement of costly chemicals and sophisticated analytical tools. With the swift transitions being made in the field of diagnostics, few polyclonal antibody-based immunochromatographic tests have been developed for detection of specific snake venoms [29]. Scientific advancements have been made to develop venom detection kits but till today none of them have been commercialised except the snake venom detection kit from Australia. Taken together the limitations being faced by clinicians and forensic experts in venom detection, we propose to provide an on-site alternative that can improve the clinical management of snakebite victims by an unambiguous identification of causative species. A recombinant venom protein based monoclonal antibody generation approach has been proposed to develop a lateral flow-based point of care diagnostic to differentially detect elapid from viper venom.

## Materials and methods

### Ethics statement

All animal experiments were conducted with due approval from the Institutional Animal Ethics Committee, National Institute of Animal Biotechnology; Sanction No.: TBPL-NIAB/02/2017

### Materials

The *Naja naja*, *Bungarus caeruleus*, *Daboia russelli* and *Echis carinatus* venoms were purchased from the IRULA Snake Catchers’ Industrial Co-operative Society, Mamallapuram, Tamil Nadu, India. All other materials have been procured from sources as specified in the course of the text.

## Methods

### Cloning, expression, and purification of recombinant cytotoxin– 7 protein (rCTX– 7)

**Fig 1:**
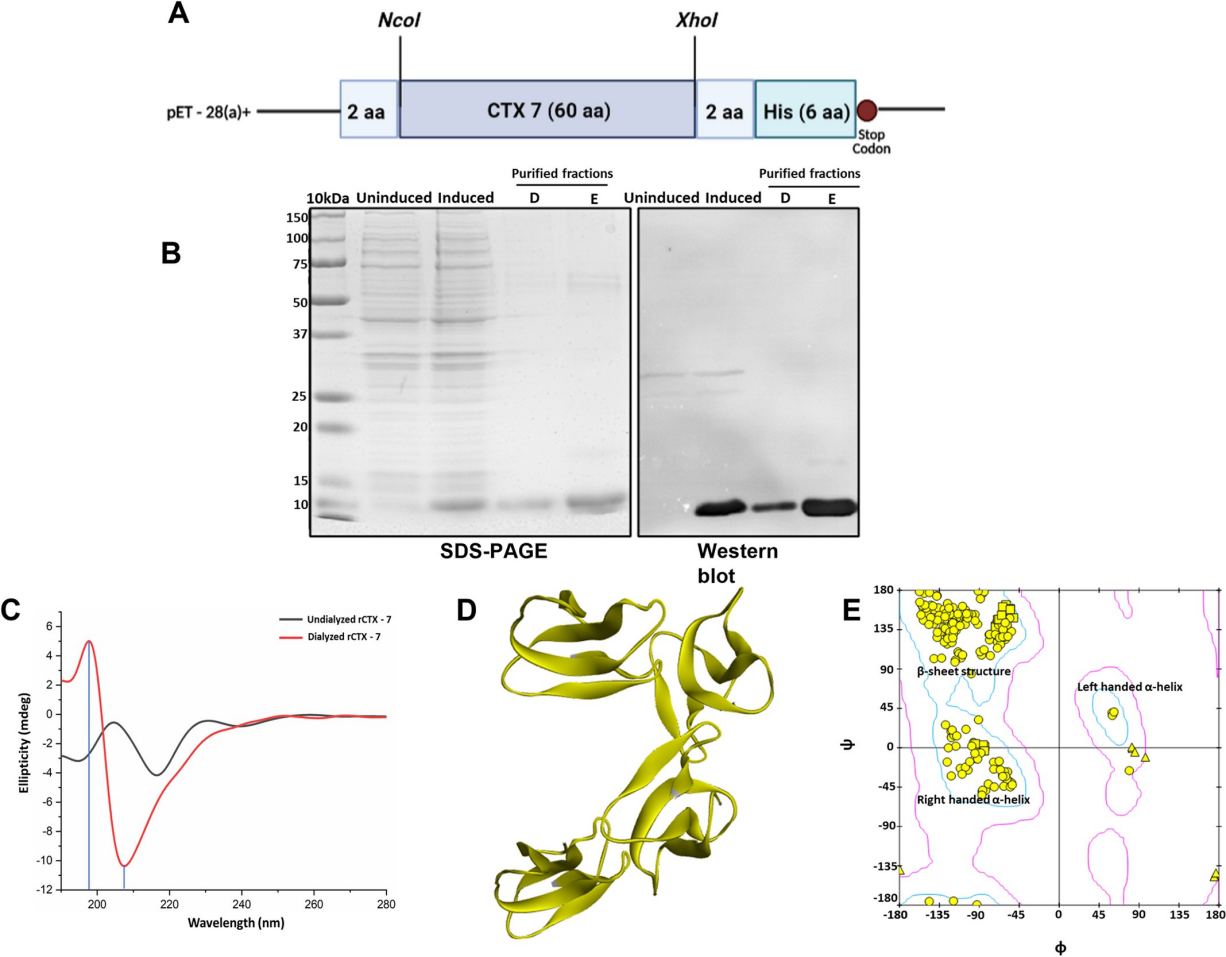
Cloning, expression, and purification of recombinant Cytotoxin-7. *Panel A*: Schematic representation of the construct showing integration of cytotoxin–7 in the pET 28a (+) vector between *Nco1* and *Xho1* restriction sites. *Panel B*: SDS–PAGE profile and Western blot analysis of induced and purified fractions of rCTX-7 from *E*. *coli* transformed cells. *Panel C*: Circular Dichroism spectrograph for undialyzed and dialyzed rCTX-7 fractions. *Panel D*: *In silico* modeling of rCTX-7 protein sequence retrieved from UniProt. *Panel E*: Ramachandran plot constructed for the rCTX-7 sequence using Discovery Studio to validate the secondary structure of the purified protein.

Based on the uniqueness, predominance, and homology with other cytotoxins of elapid venom, Cytotoxin-7 (Accession No.: P86382; CTX-7; 7.7 KDa; 60aa; three-finger toxin family) was chosen for expression as a recombinant protein. The CTX– 7 nucleotide sequence was codon-optimized for prokaryotic expression (GenScript Corp., NJ, USA). The synthetic nucleotide sequence was cloned in frame with six additional histidine residues at the C-terminus in a pET28a (+) expression vector (Fig 1A). The plasmid was transformed into *E*. *coli* BL21 DE3 competent cells using CaCl_2_ method [30], with kanamycin (30 μg/mL) resistance as the selectable marker. The transformants were subjected to plasmid isolation (Plasmid Isolation kit; QIAGEN, Hilden, Germany) followed by restriction digestion using *Xho1* and *EcoRV* restriction enzymes to confirm the presence of the desired insert in the clones. One of the positive clones was inoculated in LB broth medium and induced during the mid-log phase using the optimised concentration of isopropyl-β-D-thiogalactopyranoside (IPTG) for expression of rCTX– 7. The recombinant protein was purified by affinity–purification using Ni–NTA Agarose beads (Qiagen, Hilden, Germany). The integrity of the folded rCTX-7 protein before and after dialysis was confirmed by Circular Dichroism spectro polarimetry and the folding pattern obtained was validated by a Ramachandran plot made using BIOVIA Discovery Studio Visualizer.

### Expression and purification of tagged recombinant protein

For affinity–purification of the recombinant protein, bacterial cells were sonicated twice at 30% amplitude for 20 minutes with a pulsating frequency after resuspending it in His-Lysis buffer (NaCl; 300 mM and Tris base; 50 mM, pH 8.0) and subsequently in His-Lysis buffer containing 0.1% Triton–X 100. The sonicated bacterial cell lysate was centrifuged at 3200 x g for 30 minutes at 4°C. Finally, the pellet obtained was sonicated in a buffer (6 M Guanidine HCl and 50 mM sodium monophosphate; pH 8.0) for 5 minutes and incubated overnight at 4°C for complete solubilisation with end-to-end mixing. The solubilized suspension was centrifuged at 3200 x g for 30 minutes at 4°C and the supernatant was incubated with Ni-NTA Agarose beads (500 μL; Qiagen, Hilden, Germany) for 2 hours at 4°C for its binding with His-tag of rCTX-7. The elution was performed using varying pH (6.3, 5.9, and 4.5) buffers (8 M urea, 0.1 M sodium monophosphate, 0.01 M Tris base). The purified protein was dialyzed using a tris buffer (50 mM Tris, 1 mM EDTA, 10% Sucrose, 0.1 mM reduced glutathione, and 0.01 mM oxidized glutathione) with decreasing concentration of urea (4 M, 3 M, 2 M, 1 M, 0.5 M, and 0 M). The protein was stored in 10 mM phosphate buffer for long term storage.

### Sodium dodecyl sulphate (SDS) polyacrylamide gel electrophoresis (PAGE) and Western blot

Protein samples for electrophoresis were prepared by addition of sample buffer (Tris–HCl; 50 mM, pH 6.8, 2% SDS, 6% glycerol, 0.004% bromophenol blue, 5% β-mercaptoethanol) and heated at 95°C for 10 min and resolved by 0.1% SDS– 12% PAGE. The resolved proteins were either stained with Coomassie brilliant blue (R250; 0.1%, Sigma Aldrich, MO, USA) or transferred onto the nitrocellulose membrane using the wet transfer. After protein transfer, membranes were blocked with Tris-buffered saline (TBS; Tris–HCl 50 mM, NaCl 150 mM, pH 7.4) containing 3% bovine serum albumin (BSA; Sigma Aldrich, MO, USA) for 1 hour. Blots were incubated at 4°C overnight with 1:1000 dilution of Anti-His antibody in TBS containing 0.3% Tween-20 (TBST) and 0.1% BSA, followed by three rounds of washing with TBST and further incubation with 1:2000 dilution of horseradish peroxidase (HRP)–conjugated goat anti-mouse IgG (Invitrogen, ThermoFisher Scientific, MA, USA) in TBST for 1 hour at room temperature. The blots were washed thrice with TBST and visualised using 3, 3′ -diaminobenzidine tetrahydrochloride (DAB substrate, Roche, Sigma Aldrich, MO, USA) and imaged using Chemidoc Imaging System (Bio-Rad, CA, USA).

### Immunization of mouse with rCTX-7 protein for generation of monoclonal antibody

Three female BALB/c mice (8 weeks old; maintained at Teena Biolabs, Hyderabad, India) were immunized subcutaneously with rCTX-7 protein (50 μg) emulsified with complete Freund’s adjuvant (Sigma Aldrich, MO, USA). Mice were boosted using rCTX-7 (20 μg) emulsified in incomplete Freund’s adjuvant (Sigma Aldrich, MO, USA) through intraperitoneal route on days 14, 21, 28, and 35. The mice were bled on days 34 and 40 and the serum samples were tested for generation of humoral immune response by indirect Enzyme-Linked Immunosorbent Assay (ELISA). The mouse showing the highest antibody response was given intravenous injections on three consecutive days with un-emulsified rCTX-7(10 μg) on days 41, 42, and 43, following which the spleen was harvested to isolate the splenocytes.

### ELISA

The standard protocol for indirect ELISA was adopted with certain modifications. Briefly, each well of a 96 well microtiter plate (ThermoFisher, MA, USA) was coated with varying concentrations of synthetic peptide (P1, P2 and P3; each 20 aa long) of CTX– 7 or rCTX-7 or venom overnight at 4°C. The wells left uncoated were used as the negative control. All the wells of the microtiter plate were blocked for one hour using phosphate buffered saline (10 mM Phosphate buffer, 0.9% NaCl; PBS) containing bovine serum albumin (BSA; 1%) and Tween 20 (0.015%). Following incubation, all the wells were washed once with 10 mM PBS. Post–washing, primary antibody (different dilutions of pre-immune or immune sera/purified antibodies/cell culture supernatant) was added and incubated for an hour at 37°C. Upon completion of incubation, the wells were washed thrice with wash buffer (PBS containing 0.01% Tween 20; PBST). Antibodies bound over coated antigen were detected by the addition of peroxidase-conjugated goat anti-mouse IgG antibody (1:2000 dilution; Invitrogen, ThermoFisher Scientific, CA, USA) for 1 hour at 37°C. The unbound antibody was removed by extensive washing with PBST, and the reaction was developed using o-Phenylene-diamine (OPD; 0.5 mg/mL; Sigma Aldrich, MO, USA) containing hydrogen peroxide (0.06%; Sigma Aldrich, MO, USA) prepared in citrate-phosphate buffer (0.1 M; pH 5.0). The reaction was stopped using H_2_SO_4_ (5 N; 30 μL) and absorbance was measured at 490 nm with a reference wavelength of 630 nm using an Epoch plate reader (BioTek, Agilent Technologies, VT, US).

### Generation of hybridoma

The splenocytes harvested from the mouse with the highest antibody titer were aseptically collected and fused with myeloma (SP2/O) cells in a 2:1 ratio using polyethylene glycol (PEG; 50% v/v; Sigma Aldrich, MO, USA). The successfully fused hybridomas were selected by culturing them in a selection medium containing Hypoxanthine (100 μM)–Aminopterin (0.4 μM)–Thymidine (16 μM; HAT supplement; Sigma Aldrich, MO, USA) and foetal bovine serum (FBS; 20%; European Grade; Biological Industries, Beit HaEmek, Israel) for 10–14 days [31]. After 10 days, the clones were screened for the production of antibodies against the rCTX-7 by indirect ELISA. The clones showing high ELISA immuno-reactivity against the target antigen were selected and limiting dilution was performed to obtain a single-cell clone producing antibody against rCTX– 7. All single-cell clones were further screened for reactivity against peptides, rCTX-7, and different venoms. The hybridoma with the highest immunoreactivity was chosen for the large-scale production of monoclonal antibodies.

### Purification of monoclonal antibody and assessment of its specificity for recognition of rCTX– 7

The cell culture supernatant obtained from the hybridoma producing specific monoclonal antibody against rCTX– 7 (henceforth referred to as AB1) was clarified by centrifugation at 4000 x g for 10 minutes. It was used to purify the antibody using Protein G affinity columns following the standard protocol (GE Healthcare; IL, USA).

The specificity of the antibody was ascertained by Western blot using venom samples and ELISA with a wide array of related targets, as described previously. For further characterization of the antibody, the isotype of the purified antibody was determined using Pierce Rapid ELISA Mouse mAb Isotyping Kit (ThermoFisher Scientific, MA, USA) following the manufacturer’s instructions. Finally, the sensitivity of detection of AB1 was measured using varying concentrations of rCTX– 7 and venom proteins by Indirect ELISA.

### Binding affinity studies using Surface Plasmon Resonance (SPR)

The binding affinity of purified AB 1 towards peptide II (20aa; 21–40 aa) of CTX– 7, rCTX-7, cobra, and krait venom was determined by SPR using a Biacore 2000 optical biosensor (Biacore, Uppsala, Sweden) at 25°C. Eight hundred resonance units (RUs) of AB1 were immobilized by standard amine coupling to the surface of a research-grade CM5 chip (GE HealthCare, Uppsala, Sweden). A sensor channel immobilized with ovalbumin served as a negative control for each binding interaction. A series of different concentrations of respective proteins were passed over each channel in the running buffer (10 mM PBS; pH 7.4 with 0.05% P-20 surfactant). Both binding and dissociation events were measured at a flow rate of 30 μL/min. After every binding event, the sensor surface was regenerated by repeated washing with 4 M MgCl_2_ at a flow rate of 100 μL/min. Each binding curve was analysed after correction for non-specific binding using the signal obtained from the negative control flow channel. The kinetic parameters were obtained by fitting the data to the 1∶1 Langmuir interaction model using BIA EVALUATION 3.1 software (Biacore Life Sciences).

### Development of lateral flow assay

The lateral flow assay is a multi-segmented strip that comprises of a nitrocellulose (NC) membrane upon a backing support harbouring the absorbent pad, and a conjugate pad (mdi Membrane Technologies, PA, USA) oriented in an overlapping fashion to allow uninterrupted flow of the sample through the membrane.

#### Synthesis of Gold nanoparticles (AuNPs)

The purified AB 1 antibody was conjugated by passive adsorption onto the non–functionalized citrate–coated AuNPs (20 nm) synthesised following the methods mentioned elsewhere [32] and adopted with minor modifications. Briefly, the glassware was thoroughly washed with aqua regia and extensively rinsed with deionized water. For synthesis, 0.1 M chloroauric acid (450 μL) was added and boiled up to 200°C with continual stirring. Upon boiling, 1% sodium citrate (3 mL) was quickly added to the stirring solution leading to change in its colour from pale yellow to deep red. Once the colouration became apparent, the temperature was slowly decreased with continuous stirring for 30 minutes at room temperature. The nanoparticles synthesized were characterized using transmission electron microscopy (TEM; JEM-1400 Flash Electron Microscope, JEOL Ltd., Tokyo, Japan).

#### Preparation of gold nanoparticles (AuNPs)–antibody conjugate

For high avidity of adsorption of antibodies onto the AuNP surface, the pH of the conjugation buffer and the concentration of the antibody were titrated as described [33]. The extent of aggregation of free AuNPs was measured by recording its absorbance at 530 and 690 nm. The non-specific sites were blocked by addition of 1% BSA and incubating with end-to-end mixing for 15 minutes. The conjugated AuNPs were centrifuged at 13,000 x g for 30 minutes and resuspended in the conjugate storage buffer (Tris, 20 mM, pH 8.0; NaCl, 150 mM; BSA, 1% w/v). The photometric changes from bare AuNPs to antibody conjugated AuNPs were characterized using UV-Visible spectrophotometer (JASCO V-750 Spectrophotometer) and dynamic light scattering using the particle size analyzer (Litesizer 500, Anton Paar).

#### Conjugate pad activation

For activation, the conjugate pad was soaked into the activation buffer (20 mM PB; pH 7.3, 1% BSA, 0.25% Tween 20, 2% sucrose, and 0.02% sodium azide) and completely dried by placing it at 65°C for 2 hours.

#### Coating of safe and venom line

The immunodominant peptide II fragment of rCTX– 7 (6 μg/strip) was coated at the safe line and the goat–anti mouse IgG (H+L) antibody (0.6 μg/strip; Invitrogen; ThermoFisher Scientific, USA) was coated as the venom line. The amount of protein to be used at venom and safe line was decided by batch–to–batch optimization of conditions.

#### Assembly

All the individual components were assembled onto the NC membrane backing pad. For assessing the specificity, all the big four venoms were tested on the assay platform. The sensitivity of detection was ascertained by testing varying concentrations of the rCTX– 7 protein, cobra, and krait venom with the AuNP–antibody conjugate for its recognition at the venom line on the lateral flow assay strip. Once assembled, different venoms were spiked in 10mM PB and FBS and tested for its detection by applying them onto the kit. For every test, 20 μL of the sample was added on to the strip. The flow of the sample over the membrane was assisted by addition of 2–3 drops of 10 mM PB.

### Statistical analysis

The significance of all quantitative or semi-quantitative analyses was validated through a one–way ANOVA with Tukey’s post-analysis test using Graph Pad Prism 5 to determine the significance of binding measurements. To ascertain the limit of quantitation (LOQ) of crude venoms of *N*. *naja* and *B*. *caeruleus* in spiked samples by LFA kit a non–linear regression curve was plotted, and the values obtained were used to calculate the LOQ by [34]:

$$LOQ=\frac{3\;(SD)}{Slope}$$

where SD is the standard deviation of the non-spiked sample, and the slope is obtained from the regression curve.

## Results

### Cloning, expression, and purification of recombinant cytotoxin-7 (rCTX– 7)

For the expression of CTX-7 as a recombinant protein in the prokaryotic expression system, the gene corresponding to CTX-7 was cloned in the pET-28a (+) expression vector to transform the *E*. *coli* cells (Fig 1A). The transformants were confirmed by observing the presence of a digested fragment of ~1.5 Kb on agarose gel (S1 Fig). Expression of rCTX-7 upon induction of transformed *E*. *coli* (clone 1) cell was confirmed by observing a band of ~7 kDa on Coomassie brilliant blue-stained polyacrylamide gel and Western blotting using Anti-His Ab (Fig 1B). The recombinant protein was purified by Ni-NTA affinity chromatography, and the protein fraction eluted with buffer D (pH 5.9) and E (pH 4.5) was of the highest purity as confirmed by electrophoresis and Western blot (Fig 1B). The protein was finally dialyzed in 10 mM phosphate buffer and concentrated using a 3 KDa centrifugal membrane filter (Sigma Aldrich, MO, US). To confirm proper refolding of purified recombinant protein upon dialysis, CD spectrometry was performed. The undialyzed protein showed no defined secondary structure, however, the renatured protein (upon dialysis) showed the predominance of β pleated sheets corresponding to the peak maxima at ~ 195 nm and a minimum peak near 218 nm (Fig 1C). This folding was validated by simulating the *in silico* folding of the protein sequence (Fig 1D) and subsequently performing a Ramachandran plot (Fig 1E). Ramachandran plot confirmed that the protein has predominance of β-sheets and less of α-helices.

### Generation of hybridomas and selection of specific clones

Immune sera collected from mice immunized with rCTX-7 were checked for their immunoreactivity against rCTX-7. Upon ELISA, the mouse serum having absorbance (A_450_) > 1 at a minimum 1:1000 dilution after the 4^th^ booster was chosen for generation of hybridoma (S2 Fig). On the 10^th^ day of fusion, the culture supernatant from 16 clones showed immunoreactivity above an arbitrary cut off limit (0.3). These clones were taken for limiting dilution to get a single-cell clone. Clone (AB1) showed the highest immunoreactivity against rCTX-7 and was chosen for large scale monoclonal antibody production (S3 Fig).

**Fig 2:**
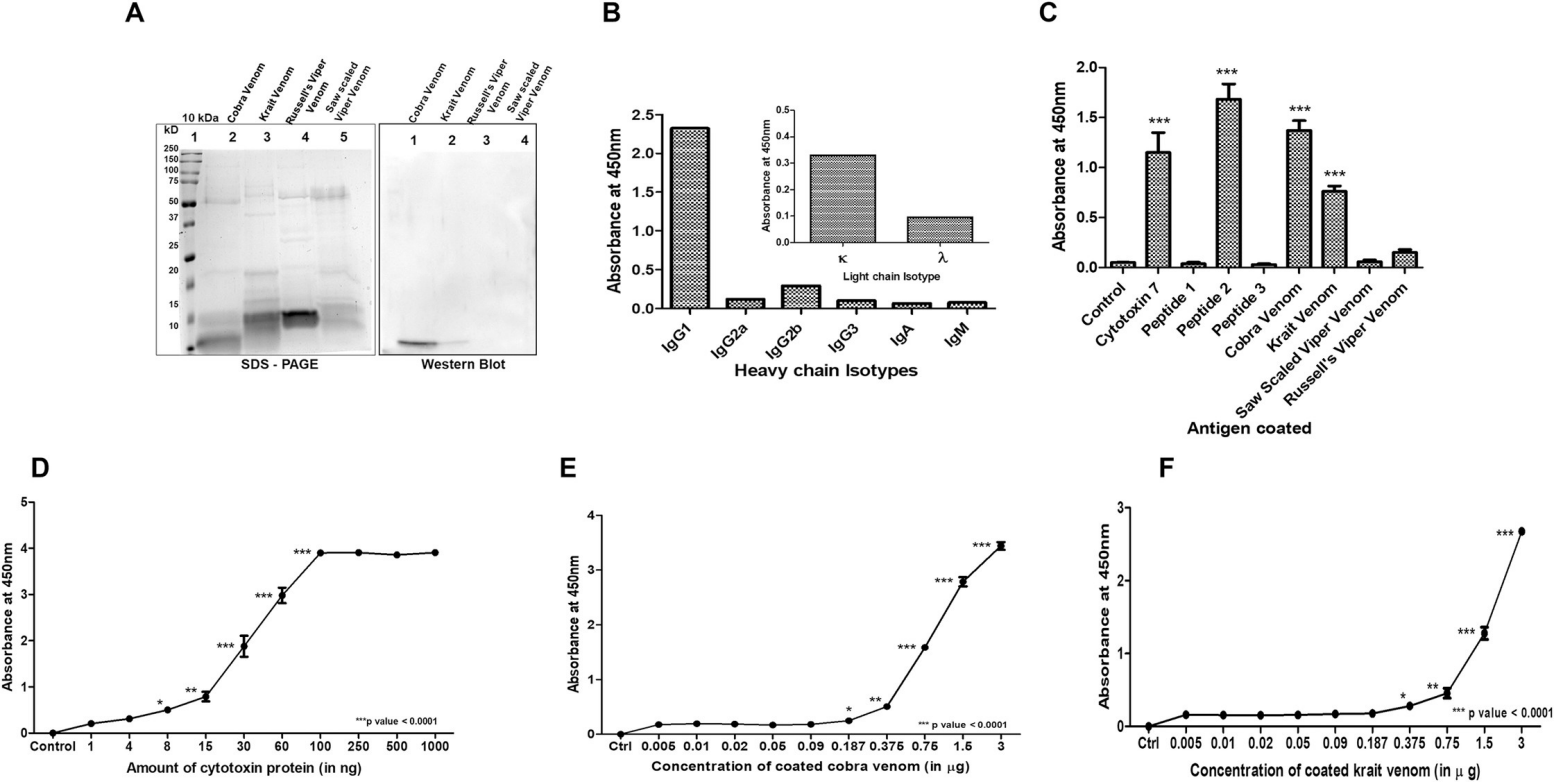
Characterization of monoclonal antibody. *Panel A*. Equal amount of venoms of big four snakes was electrophoretically separated on two gels. The Coomassie-stained gel shows the electrophoretic profile of each venom protein (5 μg) whereas the Western blot performed using monoclonal antibody shows the immunoreactivity of AB1 towards cytotoxin– 7 in respective venom samples (0.5 μg). *Panel B*. Quantitative representation of the ELISA based Isotyping of the heavy and light chain fragments of the purified antibody (AB1). *Panel C*. The graph depicts the reactivity of different antigens with AB1 monoclonal antibody. The data shows the Mean ±SE of 3 different experiments (in duplicates). The *y—axis* shows the absorbance values obtained for each antigen after normalization with the values obtained for uncoated control. *Panel D*, *E*, *and F*. Varying concentrations of rCTX-7, cobra, and krait venom were tested to determine the sensitivity of its detection by AB1 through indirect ELISA. The *y–axis* shows the mean absorbance ±SE of three different experiments performed in duplicates after normalization with the values obtained for uncoated wells. The statistical significance of each sample was calculated with respect to control, unless otherwise indicated in the figure (***p < 0.0001).

To check the specificity of AB1 towards big four snake venoms, an equal amount (5 μg for SDS-PAGE and 0.5 μg for western blot) of each was electrophoretically separated on a 0.1% SDS-12% polyacrylamide gel. Upon Coomassie blue staining, several proteins of varying molecular weight were observed in wells loaded with different venoms (Fig 2A). However, upon Western blotting using AB1 primary antibody, only one band of ~ 7 KDa was observed in cobra and krait venom but not in viper venoms. Further, AB1 antibody was isotyped as IgG_1_ heavy chain and κ light chain (Fig 2B). The affinity purified AB1 antibody showed significantly higher immunoreactivity upon ELISA with rCTX-7 (p < 0.0001) and the synthetic peptide II of CTX-7 (p < 0.0001; Fig 2C). However, there was no significant immunoreactivity of the AB1 antibody against peptide I and peptide III of CTX-7. As compared to both viper venoms, AB1 showed significantly higher immunoreactivity against cobra and krait venoms. There was no significant difference in immunoreactivity of AB1 towards rCTX-7, peptide II of CTX-7, and cobra venom. Krait venom showed significantly lower immunoreactivity than cobra venom (Fig 2C).

Indirect ELISA was performed to check the extent of detection of rCTX-7, cobra, and krait venom by AB1 antibody. The dynamic range for detection of rCTX-7, cobra, and krait venom by AB1 was 10–100 ng, 0.1–3 μg, and 0.5–3 μg (Fig 2D–2F).

### Surface Plasmon Resonance (SPR) analysis for the determination of binding affinity of AB1 to specific targets

**Fig 3:**
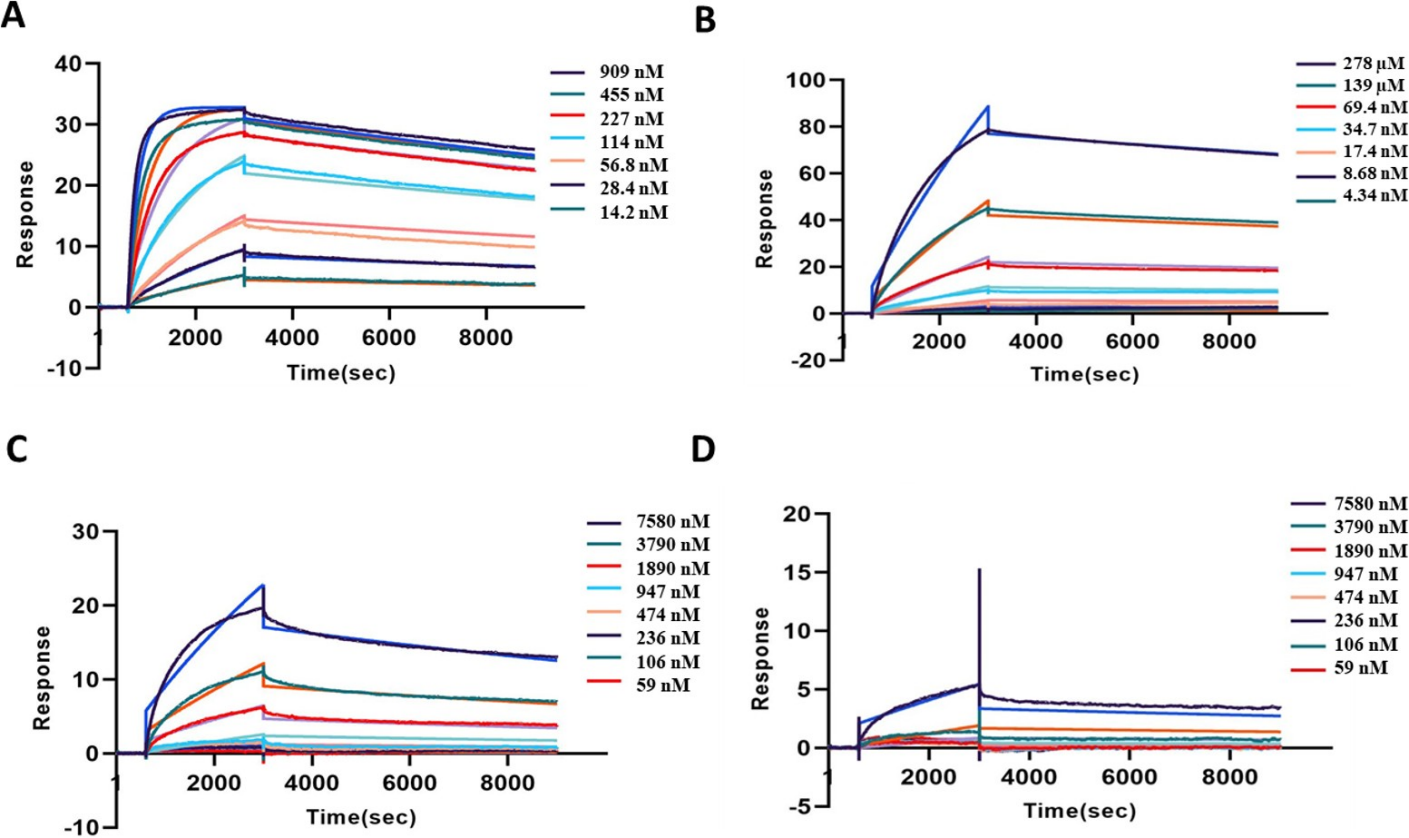
SPR Sensograms to measure the binding affinity of AB1 towards venom components. *Panels A to D*. Different concentrations of peptide-II of CTX-7, rCTX-7, cobra venom, and krait venom were taken as indicated and change in the corresponding sensograms were recorded using Surface Plasmon Resonance, respectively.

Sensograms of different samples (peptide–II, rCTX– 7, cobra, and krait venom) were recorded following interaction of analytes with the AB1 antibody immobilized on sensor chip for analysis of binding kinetics. The experiment was performed in duplicates and the dissociation constants of peptide II, rCTX-7, cobra, and krait venoms with AB1 were estimated to be 7.37 nM, 31 nM, 311 nM and 149 μM, respectively (Fig 3).

### Development of monoclonal antibody (AB 1) based lateral flow-based detection system

**Fig 4:**
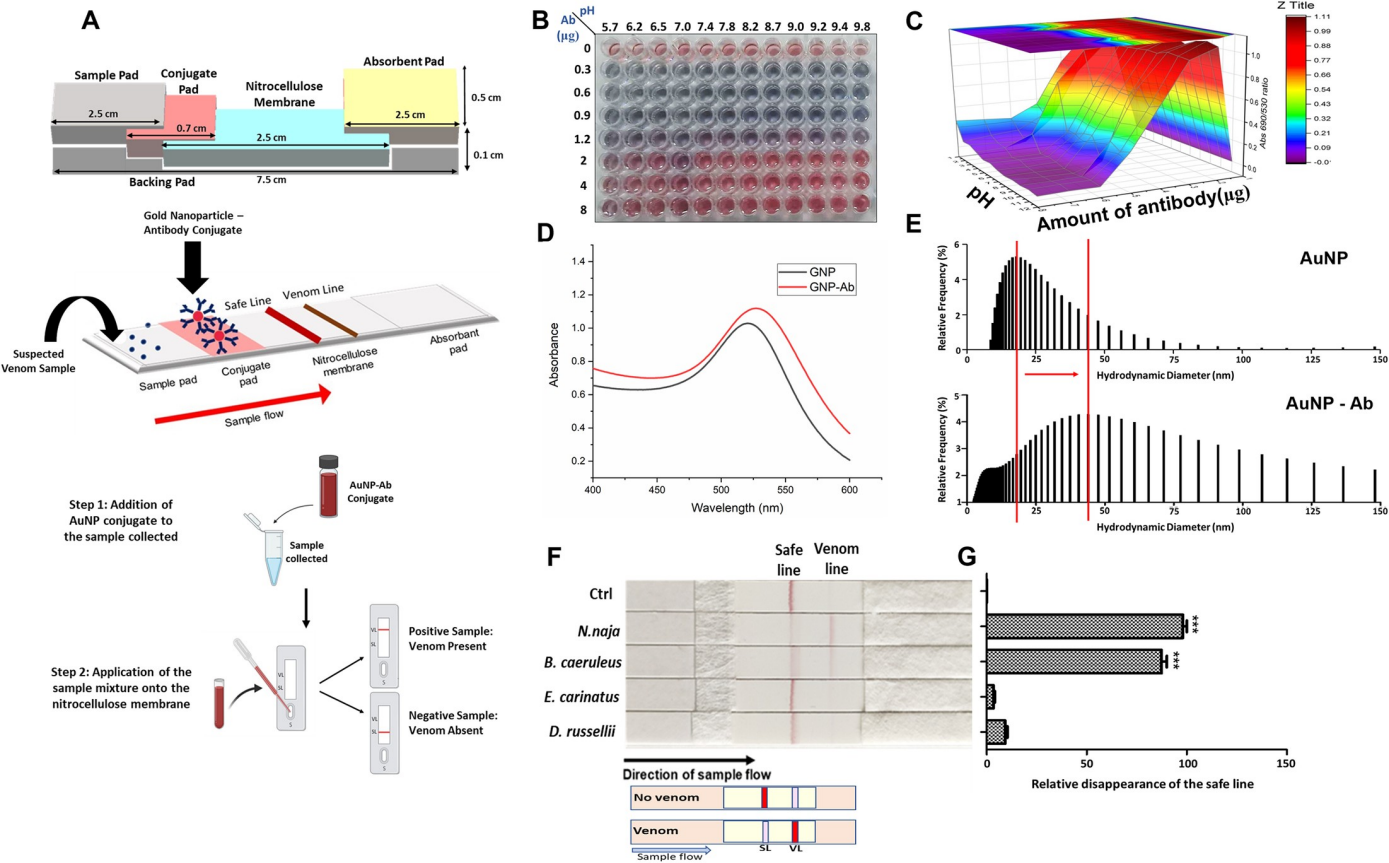
Development of the lateral flow assay kit. *Panel A*. The schematic representation seeks to explicate the workflow for testing venom sample on the LFA kit and emphasises on the various components involved, their dimensions, and orientation as in the developed LFA kit. The site of application of sample and the direction of flow is as indicated. *Panel B*. 96-well microtiter plate depicting characteristic colour change from unaggregated to aggregated AuNP while optimization of AuNP-Ab conjugation parameters. *Panel C*. 3D graph depicting the quantitive analysis of A_690_/A_530_. *Panel D*. UV-Visible spectrograph depicting a red shift in maximum absorbance of bare AuNP upon conjugation. *Panel E*. A graphical representation of the change in relative frequency distribution (%) of AuNP size before and after conjugation with monoclonal antibody (AB1). *Panel F*. The lateral flow assay strip was developed, and spiked venom (3 μg) was applied on each strip to check the specificity of venom detection. *Panel G*. Quantitative analysis for venom detection was done by densitometry using ImageJ software. The mean ± SE of the safe line density from three different experiments was calculated for each sample. The graph shows the relative disappearance of the safe line as compared to the control. Each sample was statistically analysed with respect to control. The efficiency of binding was ascertained by the statistical significance of the data obtained as is indicated in the graph (***p< 0.0001).

A schematic workflow to depict different components of LFA and its working principle has been summarized in Fig 4A. Gold nanoparticles (20 nm; S4 Fig) were conjugated with monoclonal antibody at varying concentration and pH (Fig 4B). The wells showing A_690_/A_530_ closest to zero were considered as the optimum conditions for conjugation (Fig 4B and 4C). For large scale conjugate preparation, 40 μg of AB1 was added per mL of AuNPs (OD: 1) at pH 9.4. AuNP-Ab conjugate showed a red shift in peak absorbance as compared to bare AuNPs (Fig 4D). A relative shift in the size distribution frequency (%) from 20 to 40 nm was observed for AuNP-Ab conjugate as compared to AuNPs (Fig 4E).

For development of lateral flow based differential detection platform; at an optimised condition, the safe line was coated with 6 μg of peptide–II of rCTX-7 while venom line was coated with 0.6 μg of goat anti mouse IgG antibody (Invitrogen, ThermoFisher Scientific, CA, USA) on a 200 CNPH nitrocellulose membrane (Advanced Microdevices, Ambala Cantt., India). For validation of specific detection, big four venoms (3 μg) were spiked into the 10 mM Phosphate buffer keeping a no–venom buffer sample as a negative control. Application of cobra and krait venom spiked samples onto the test strip of the kit led to a significant (p < 0.0001) disappearance of safe line as compared to control where venom was not added (Fig 4F). There was no significant disappearance of safe line with venoms of Russell’s viper or saw-scaled viper (Fig 4F). The significance of this binding was ascertained by a quantitative analysis of the relative disappearance of the safe line in each sample as compared to no-venom control (Fig 4G).

**Fig 5:**
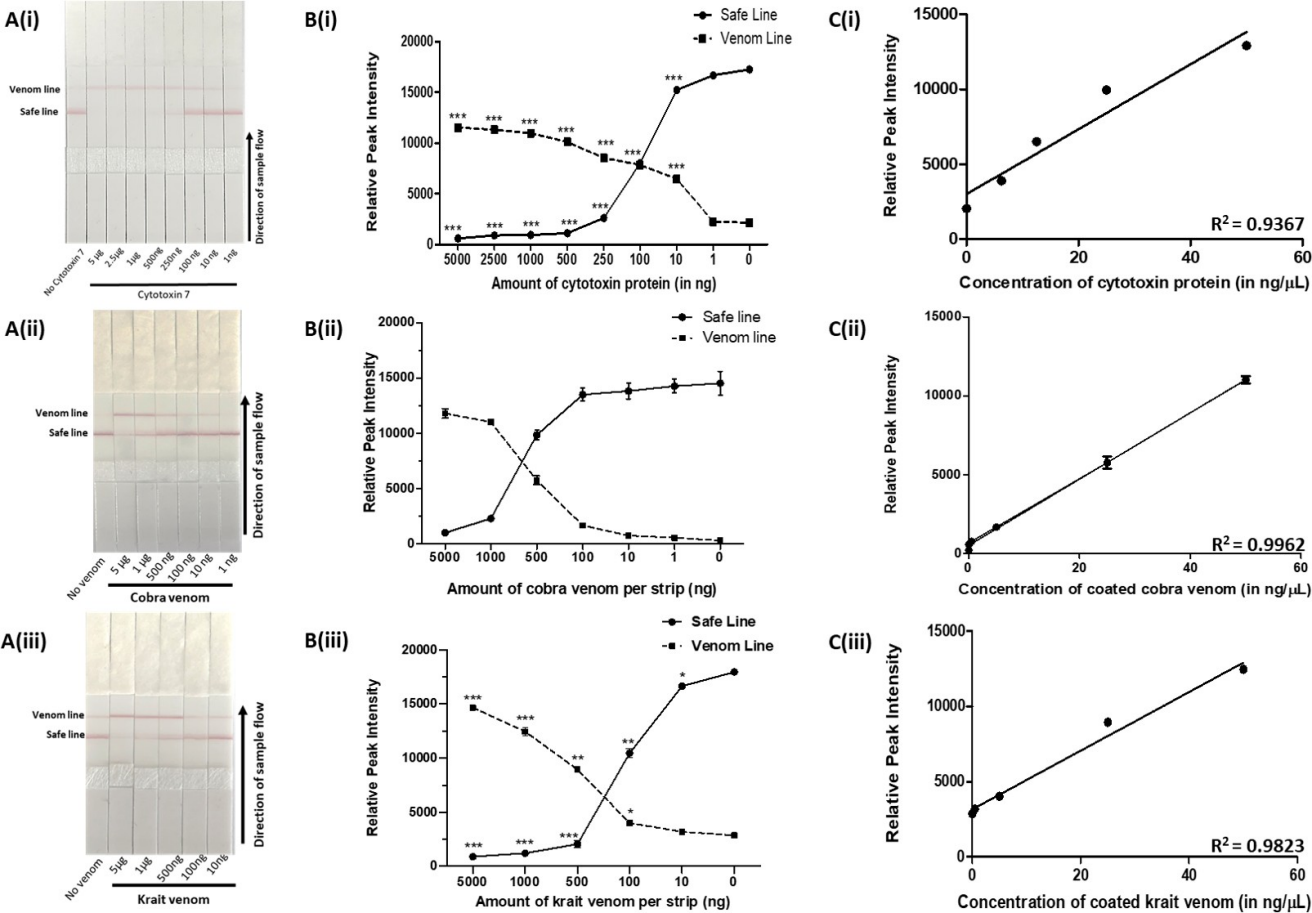
Limit of detection of venoms of *N*. *naja* and *B*. *caeruleus* by AB1 using the lateral flow assay kit. *Panel A*. Varying amounts of spiked venom samples were applied onto the strip of the LFA kit and the disappearance of safe line was noted. *Panel B*. Densitometric analysis of each strip was done using ImageJ and statistical analysis was done for each sample with respect to control (***p < 0.0001). The relative intensities were plotted in a line curve using GraphPad Prism 5. *Panel C*. The data points encompassing the linear range of detection of respective analytes were fit in a straight line for a non–linear regression curve to determine the limit of quantitation.

**Fig 6:**
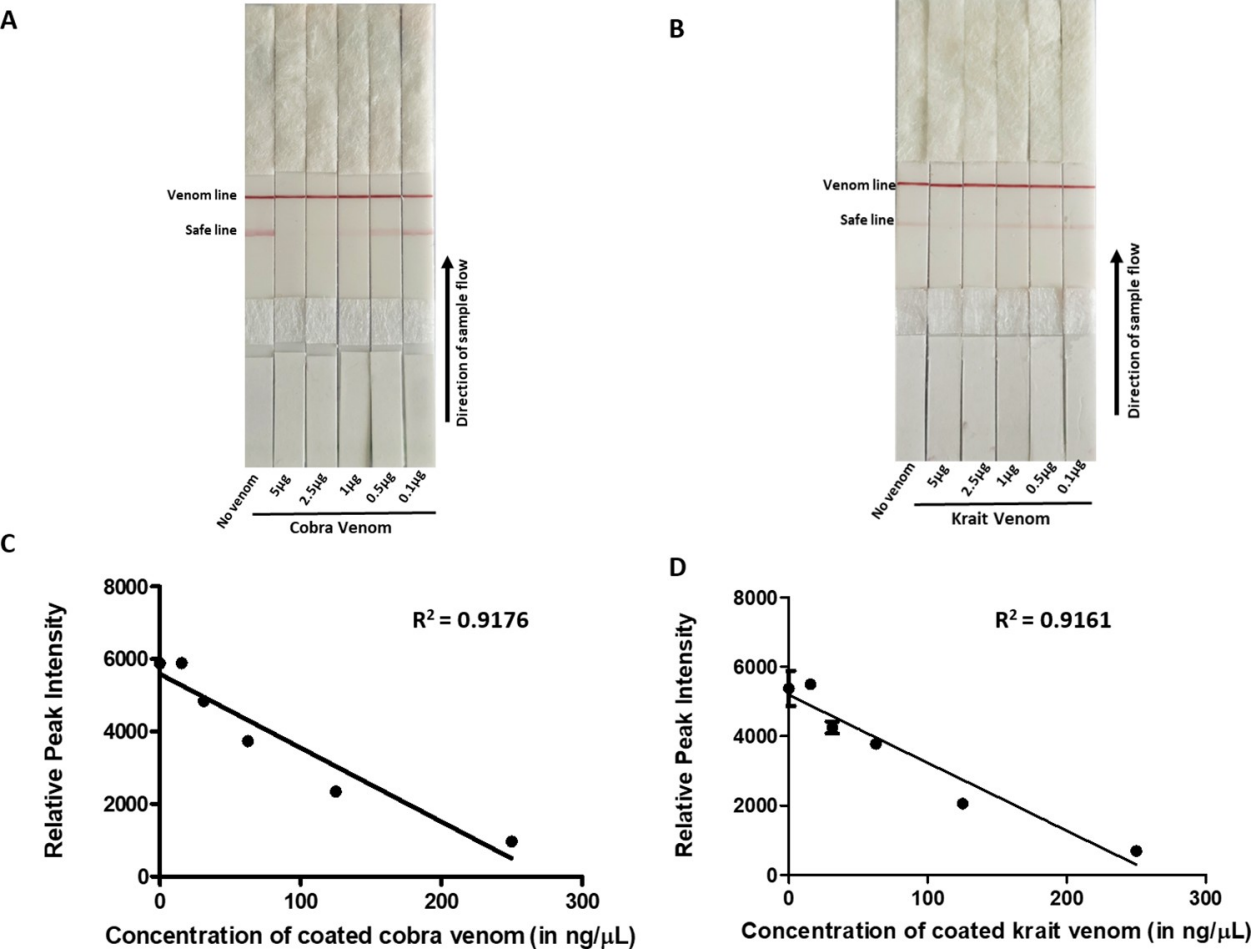
Detection of venoms of *N*. *naja* and *B*. *caeruleus* by the kit in spiked serum samples. *Panel A and B*. Varying amount of venom samples spiked into serum were applied onto the strip of the LFA kit and the disappearance of safe line was noted. *Panel C and D*. The data points obtained from densitometric analysis of safe line were fit in a straight line for a non–linear regression curve using effective venom concentration to determine the limit of quantitation.

To determine the sensitivity of detection of rCTX-7, cobra and krait venom by the lateral flow assay, different concentrations of the rCTX-7 (5 μg– 1 ng), cobra venom (5 μg– 1 ng) and krait venom (5 μg– 10 ng) were spiked in buffer and serum. In the samples where rCTX-7 and/or venom was spiked in buffer, there was a concentration-dependent decrease in safe line (Fig 5A). A naked eye analysis of the data revealed that the LFA kit has a limit of detection in the range of 250 to 5 ng/μL, 250 to 25 ng/μL, and 250 to 25 ng/μL for rCTX-7, cobra venom, and krait venom respectively in a total sample volume of 20 μL applied onto the test strip (Fig 5B). Semi-quantitative analysis of the data was performed to analyse the limit of quantitation (LoQ) (as mentioned in the *Materials and Methods*). We observed that the LFA kit has LoQ of 170 pg/μL for rCTX-7 and cobra venom, and 2.1 ng/μL for krait venom (Fig 5C). The effectiveness of detection of the venom samples by the kit was tested in a complex matrix by spiking different concentrations of cobra and krait venom (5 μg– 0.1 μg) in foetal bovine serum (Fig 6A and 6B). The LoQ of the kit for detection of cobra and krait venom in serum was calculated as 28.7 ng/μL and 110 ng/μL, respectively (Fig 6C and 6D).

## Discussion

Identification of suitable biomarkers is the bottleneck for differential detection of any venom or toxin. For developing a field-level molecular diagnostic, the biomarker(s) should be unique and abundantly present in the venom sample, irrespective of the geo-climatic conditions. Till date, there are no reports of venom specific biomarkers for differential diagnosis of snake bite [35]. Except for the snake venom detection kit (SVDK) from Australia (for indigenous snakes), none of the serological or immunological tests have been commercialized for field utilization due to varied reasons [36]. Despite the evolutionary changes arising in the genomic and proteomic composition of venoms, multiple protein families have been reported to be ubiquitously present in several species across variable topographical locations, making species-specific identification a challenge [27,31]. Data mining across the proteomic databases for different snakes of the elapid family (majorly, *Naja sp*. and *Bungarus sp*.) led us to identify cytotoxin-7 as a biomolecule for differential detection of elapid venom from viperidae of the big four snake venoms [37].

Cytotoxins are predominant protein of three-finger toxin family which are expressed in several isoforms. Cytotoxins make up ~ 40–70% of the whole cobra venom proteome [38] whereas, the three-finger toxins in the krait venom proteome make up ~20% of the whole protein composition [39]. It has also been observed that its level does not exhibit much geographical variation. Although the relative abundance of cytotoxin proteins is variable between the Indian cobra and the Indian krait; the immunodominant synthetic peptide II of rCTX–7 shares sequence homology with most of the other isoforms of cytotoxin present in elapid venom (S5 Fig). The presence of cytotoxin-7 has not been found in the venoms of the viperidae or crotalinae family [40,41]. Furthermore, CTX–7 shares no sequence homology with the human genome. The relative abundance and specificity of cytotoxins for elapid venom make it an ideal biomarker for differential detection with other venoms of big four snakes.

Full-length codon optimized cytotoxin- 7 protein with six histidine residues at the C- terminus was expressed as ~ 7 kDa protein in *E*. *coli* (Fig 1). Prior to animal experiments, protein refolding and its conformation was verified through circular dichroism spectro-polarimetry and Ramachandran plot. The renatured protein showed characteristic curves for beta rich protein which was in concordance with the theoretical prediction (Fig 1).

The highest reactivity of the purified antibody was observed with the region (peptide-II) which is conserved across different forms of cytotoxins present in elapid venom (S5 Fig). Both immunoreactivity, as well as binding affinity, was observed to be highest with peptide-II and relatively lesser for rCTX-7, cobra and krait venom. A significantly lower immunoreactivity and binding affinity towards krait venom as compared to cobra venom may be attributed to the lower level of expression of cytotoxins in krait venom (Fig 2B). Aptamers have been selected against Phospholipase A2 for the detection of krait venom [42,43]. Even so, the universal presence of this protein in both elapid and viper venoms raise questions on its utility as a marker for species-specific venom identification. Polyclonal antibody-based enzymatic- or radioactivity-assays can detect venom up to 1–5 ng/mL in the sample [28,44]. These assays require long detection time and expertise which is undesirable for rapid point-of-care testing of envenomation. Apart from the possibility of cross-specificity with other venoms, the polyclonal antibody-based assays also hold the limitation of batch-to-batch variation in commercial production.

LFA is proving to be successful for field-level rapid detection of various diseases [45]. Its ease of production at low cost has made it a valuable diagnostic method in resource limiting settings. Recently, a polyclonal antibody based immunochromatographic test has been developed for detection of various species of cobra venom with a detection limit of 5 ng/mL [29]. But there is no substantial data for its application as a kit for differential diagnosis of envenomation. For designing the lateral flow assay, optimized concentration of cytotoxin peptide-II and goat anti-mouse IgG antibody was coated in the form of the fine line at the safe and venom line (Fig 4). The LFA has been designed in such a way that upon application of sample (spiked with venom) there will be competitive displacement of antibody at safe line. The antibody-venom protein complex moving ahead from the safe line will get captured at the venom line by anti-IgG antibody. Disappearance of a red line at safe line of NC membrane is confirmatory for the presence of elapid venom in the sample.

An adult cobra can release ~110 mg of venom (in dry weight) in a single yield [45] whereas up to 115 mg of dry weight krait venom has been listed for a single bite [46]. The LD_50_ for cobra and krait venom in mouse has been estimated to be 1.91 mg.kg^-1^ and 0.1 mg.kg^-1^ body weight respectively [3,47]. Therefore, the limit of quantitation of 28.7 ng/μL for cobra and 110 ng/μL of krait venom attained by the developed assay may find utility to detect elapid venom even in cases of partial bites (Fig 6). LFA based differential detection of elapid venom from the venom of big four snakes without cumbersome sample preparation and prolonged detection time will pave the way for precise antivenom therapy. Furthermore, the methods followed to develop this kit can be used to fabricate such assays for other venoms.

In future, development of a quantitative biosensor using such monoclonal antibodies may overcome the limitations of LFA to facilitate real time monitoring of envenomation in patients. Moreover, the sensitivity of LFA kit can be further enhanced by optimization of the conjugate loading pad and the absorbent pad with higher sample holding capacity. The ability of various membrane components to carry higher sample volume will facilitate detection of venom in forensically challenged and highly diluted samples. Further studies can also be employed for exploring the detection capabilities of the kit in geographically variable species’ samples. Venom specific monoclonal antibody may also be explored for its therapeutic potential and venom neutralization abilities.

## Conclusion

Cytotoxin– 7 has been identified as a potential biomarker for differential detection of elapids venom from that of viperidae. AB1 monoclonal antibody generated against rCTX-7 has been used for the development of a lateral flow-based venom detection kit for differential detection of *Naja naja* and *Bungarus caeruleus* bites from vipers of big four snakes in buffer as well as in serum. The kit will aid in visual detection of venoms of *N. naja* and *B. caeruleus*; which might prove to be an integral tool in undertaking wide-scale field testing of envenomation cases.

## Supporting information

S1 Fig Agarose Gel Electrophoresis depicting the positive clones with a fallout of ~1.5Kb. (TIF)

S2 Fig The polyclonal immune response of mouse after 3rd and 4th booster of rCTX– 7 was analyzed by indirect ELISA at different dilutions of the serum sample. (TIF)

S3 Fig Differences in the immunoreactivity of antibody produced by sixteen different clones after hybridoma generation was determined by indirect ELISA using the culture supernatant as the primary antibody. (TIF)

S4 Fig Transmission Electron Microscopy (TEM) image of the citrate coated gold nanoparticles (AuNPs). (TIF)

S5 Fig Multiple Sequence Alignment of the synthetic peptide II (CTX-PEP2) sequence with other isoforms of Cytotoxin proteins present in venom. (TIF)
