# Supplementary figures and images for: Cytotoxin antibody-based colourimetric sensor for field-level differential detection of elapid among big four snake venom
Source: PLoS Negl Trop Dis. 2021 Oct 11;15(10):e0009841. doi: 10.1371/journal.pntd.0009841 (PMC8530336; doi:10.1371/journal.pntd.0009841)

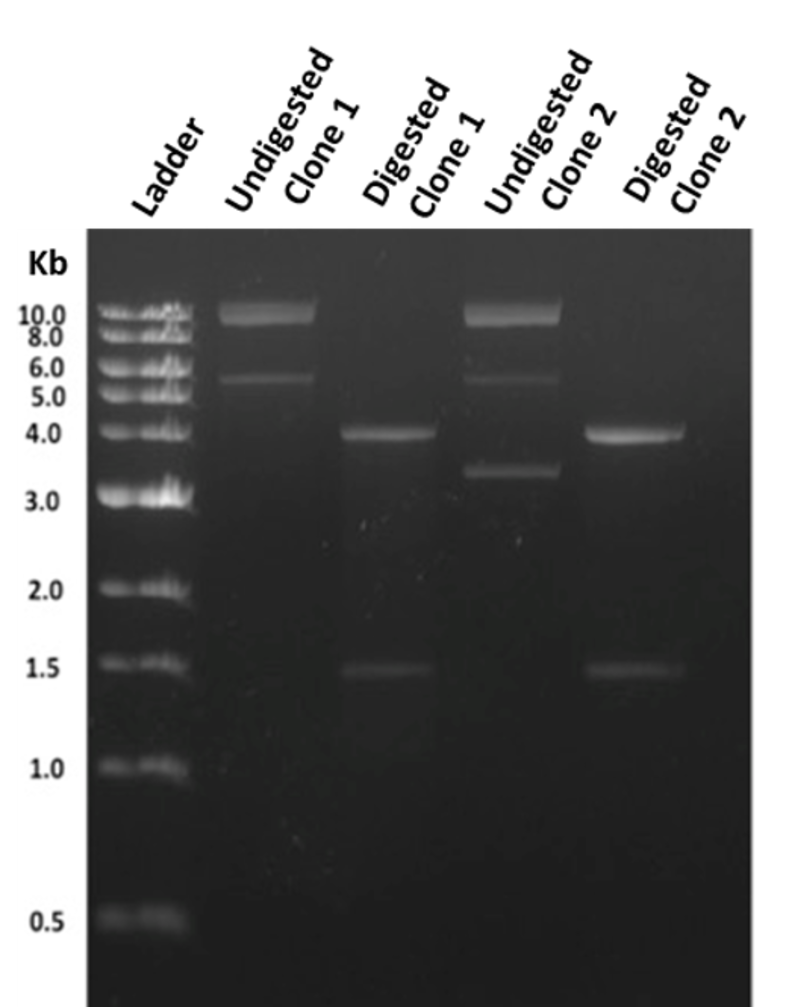

Supplement: S1 Fig — (TIF) [file pntd.0009841.s001.tif]

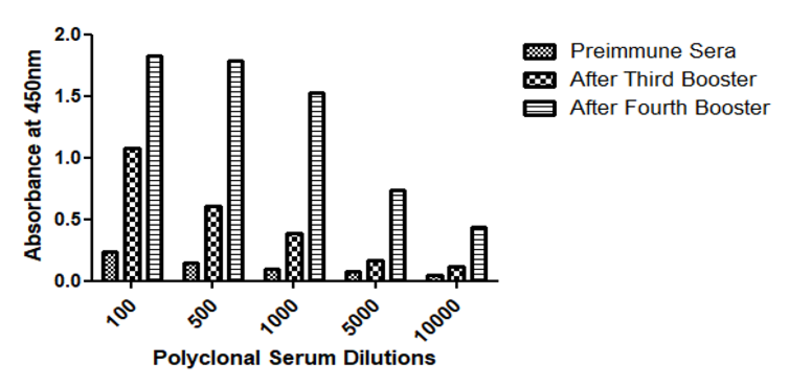

Supplement: S2 Fig — (TIF) [file pntd.0009841.s002.tif]

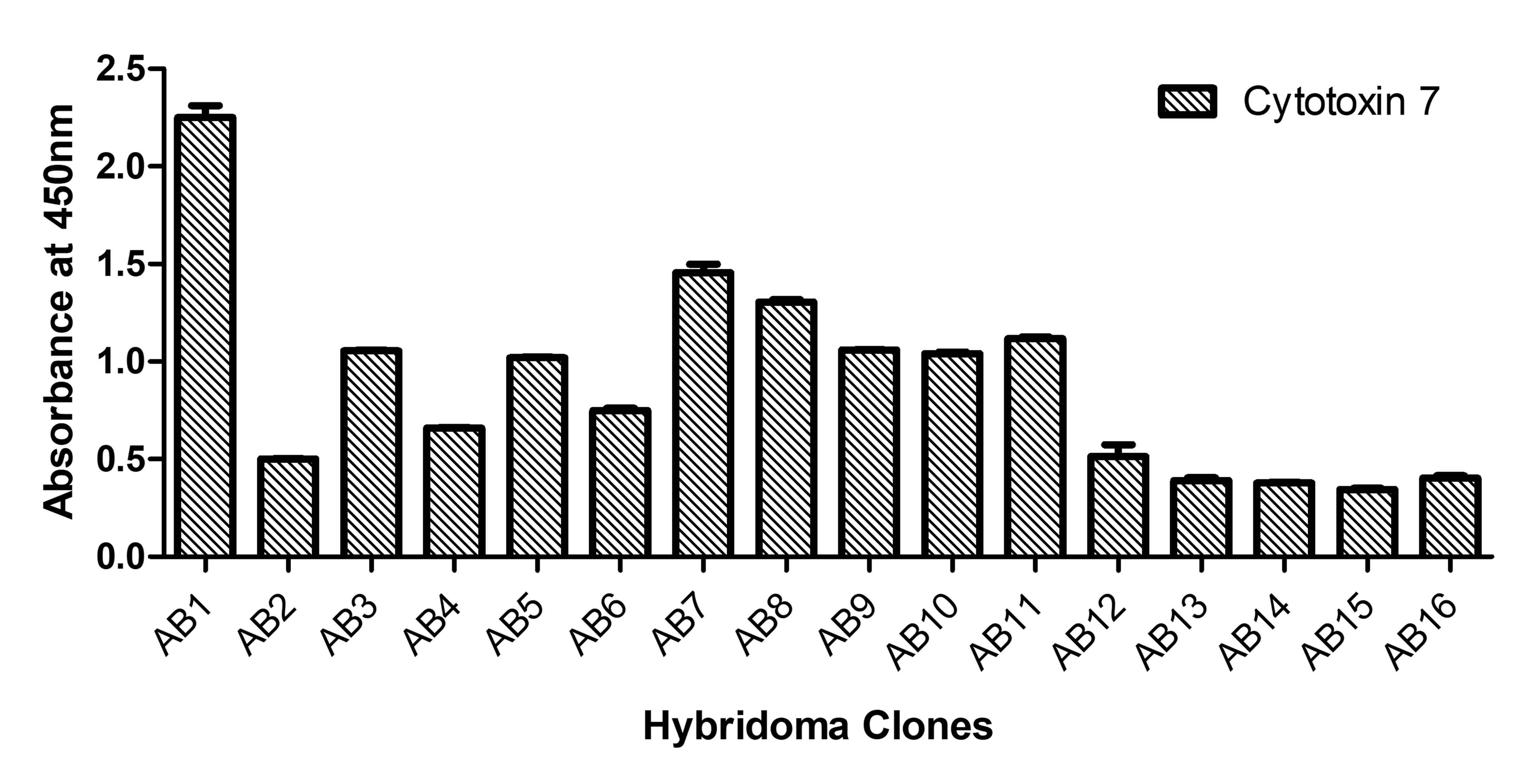

Supplement: S3 Fig — (TIF) [file pntd.0009841.s003.tif]

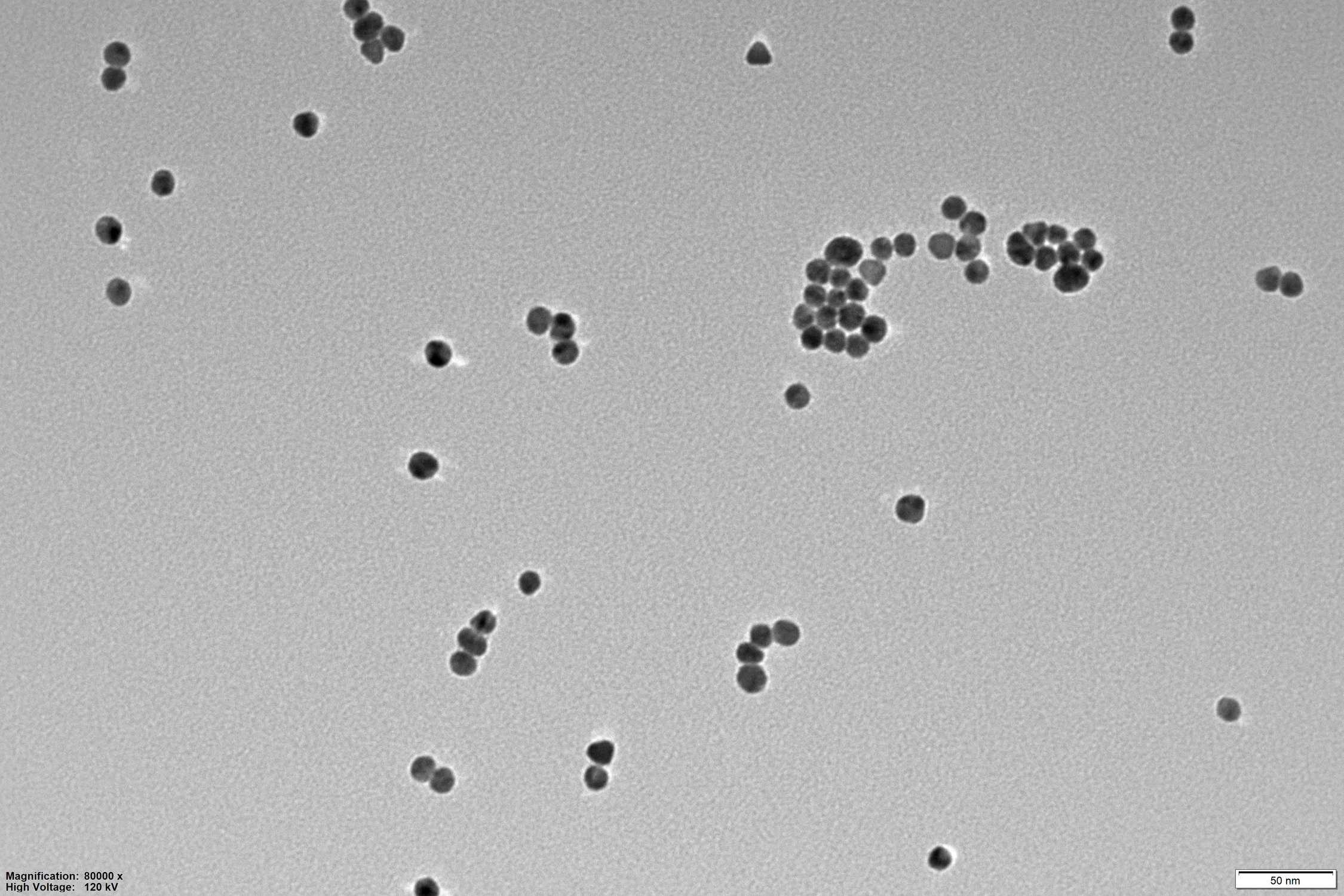

Supplement: S4 Fig — (TIF) [file pntd.0009841.s004.tif]

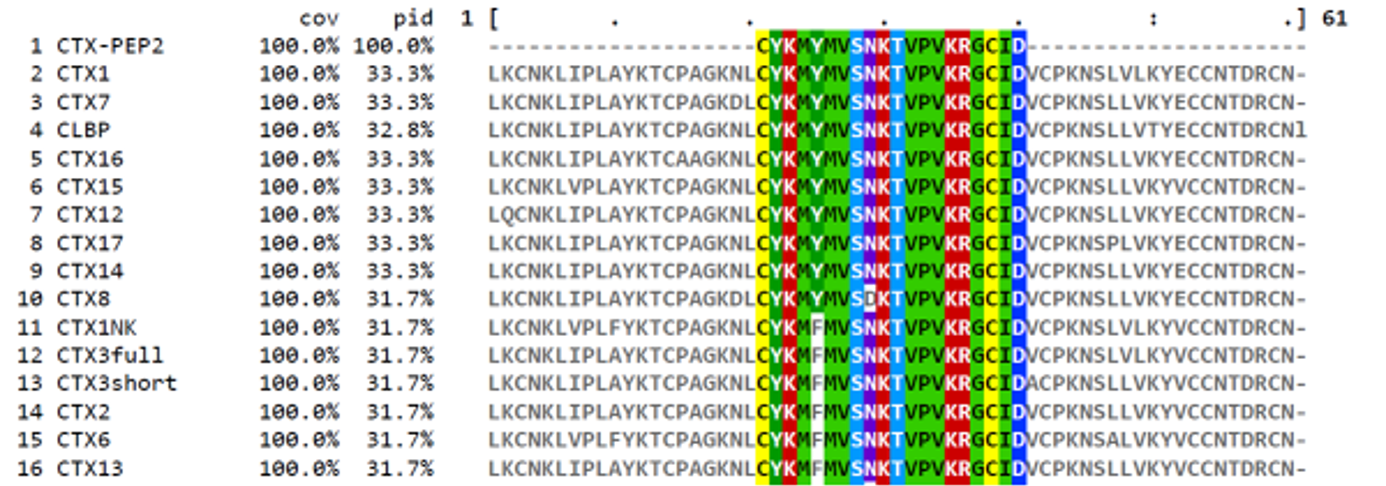

Supplement: S5 Fig — (TIF) [file pntd.0009841.s005.tif]
